# Chalcogen Bonding in the Molecular Dimers of WCh_2_ (Ch = S, Se, Te): On the Basic Understanding of the Local Interfacial and Interlayer Bonding Environment in 2D Layered Tungsten Dichalcogenides

**DOI:** 10.3390/ijms23031263

**Published:** 2022-01-23

**Authors:** Pradeep R. Varadwaj, Arpita Varadwaj, Helder M. Marques, Koichi Yamashita

**Affiliations:** 1 Molecular Sciences Institute, School of Chemistry, University of the Witwatersrand, Johannesburg 2050, South Africa; Helder.Marques@wits.ac.za; 2 Department of Chemical System Engineering, School of Engineering, The University of Tokyo, 7-3-1, Tokyo 113-8656, Japan; varadwaj.arpita@gmail.com (A.V.); yamasita@chemsys.t.u-tokyo.ac.jp (K.Y.)

**Keywords:** tungsten dichalcogenide dimers, chalcogen bonding, energy stability, natural orbital-based characterizations, MESP-, LOL-, QTAIM-, RDG-, IGM-, and IRI-based analyses

## Abstract

Layered two-dimensional transition metal dichalcogenides and their heterostructures are of current interest, owing to the diversity of their applications in many areas of materials nanoscience and technologies. With this in mind, we have examined the three molecular dimers of the tungsten dichalcogenide series, (WCh_2_)_2_ (Ch = S, Se, Te), using density functional theory to provide insight into which interactions, and their specific characteristics, are responsible for the interfacial/interlayer region in the room temperature 2H phase of WCh_2_ crystals. Our calculations at various levels of theory suggested that the Te···Te chalcogen bonding in (WTe_2_)_2_ is weak, whereas the Se···Se and S···S bonding interactions in (WSe_2_)_2_ and (WS_2_)_2_, respectively, are of the van der Waals type. The presence and character of Ch···Ch chalcogen bonding interactions in the dimers of (WCh_2_)_2_ are examined with a number of theoretical approaches and discussed, including charge-density-based approaches, such as the quantum theory of atoms in molecules, interaction region indicator, independent gradient model, and reduced density gradient non-covalent index approaches. The charge-density-based topological features are shown to be concordant with the results that originate from the extrema of potential on the electrostatic surfaces of WCh_2_ monomers. A natural bond orbital analysis has enabled us to suggest a number of weak hyperconjugative charge transfer interactions between the interacting monomers that are responsible for the geometry of the (WCh_2_)_2_ dimers at equilibrium. In addition to other features, we demonstrate that there is no so-called van der Waals gap between the monolayers in two-dimensional layered transition metal tungsten dichalcogenides, which are gapless, and that the (WCh_2_)_2_ dimers may be prototypes for a basic understanding of the physical chemistry of the chemical bonding environments associated with the local interfacial/interlayer regions in layered 2H-WCh_2_ nanoscale systems.

## 1. Introduction

Group VI transition metal dichalcogenides (TMDs or TMDCs) are a class of layered materials discovered during the last century [1,2,3,4]. Due to the fact that they find application in a wide range of technological areas, they have been the focus of intense research more recently (for example, [5,6,7,8,9,10]). Stable TMDCs (approximately 60 in number) have the generic formula AB_2_, [11,12], where A is a transition metal and B is chalcogen (S, Se, Te). These materials are sometimes described with formulae such as ME_2_, MX_2_, and MCh_2_ (M = transition metal and X, Ch, E = chalcogen derivative) [3,13,14,15], among others. TMDCs that have been particularly intensively studied are the dichalcogenides of W and Mo [3,16,17].

**Figure 1:**
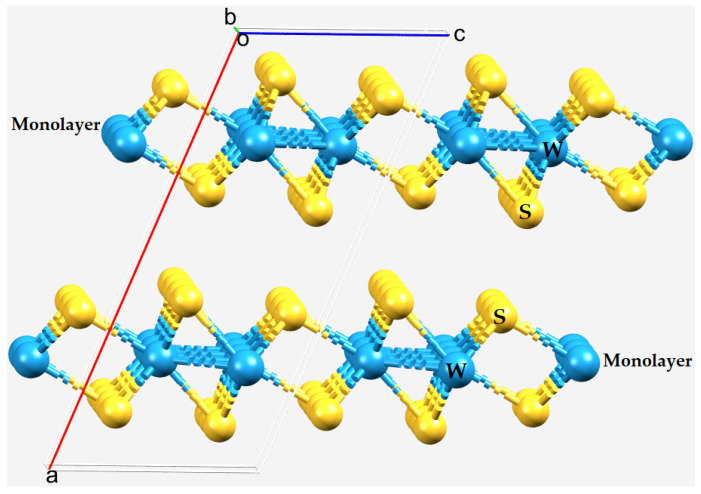
The bilayer structure of 2H-WS_2_ (651384–ICSD [2]) viewed with the *b*-axis (slightly offset) perpendicular to the page. Atom type is shown.

In layered AB_2_ structures, each B–A–B monolayer comprises three atomic layers, with the transition metal (A) layer sandwiched between two chalcogen layers (Figure 1). The gap between two monolayers is referred to as the van der Waals gap [18,19], an interlayer gap within which the metal-bonded chalcogenides of the two monolayers interact non-covalently to hold the structure together [15,20,21,22]. The weak interactions between the layers allow for their mechanical exfoliation [18]. Simply referring to the interaction between the monolayers as being due to weak van der Waals forces may be simplistic and overlooks the actual nature of the interaction between the dichalcogenides of the neighboring monolayers.

Interest in group VI TMDCs stems from their potential to display marked anisotropic physical properties, including intercalation akin to other layered materials, such as graphene and silicate clay minerals, and an indirect-to-direct bandgap crossover when the monolayer is exposed to bright photoluminescence at room temperature [15,23], as well as for the valley degree of freedom associated with the non-centrosymmetric semiconducting phase [24]. TMDC monolayers behave differently to bulk materials that feature an indirect bandgap. For instance, the monolayer and bulk forms of MoS_2_ display a direct and an indirect bandgap of 1.80 and 1.29 eV, respectively [25]; both the character and the magnitude of the bandgap changes significantly when passing from the monolayer to the bulk form. 

As the number of layers decreases, the material exhibits different optical characteristics. In particular, MoSe_2_ has a crossover from an indirect bandgap of 1.1 eV to a direct bandgap of 1.55 eV [26] on passing from a bilayer to a monolayer. Similarly, WS_2_ shows a crossover from an indirect bandgap of 1.4 eV to a direct bandgap transition of 2.1 eV [27]. Clearly, these are superior materials compared to metallic graphene [28], insofar as they exist in different phases depending on the temperature and pressure and feature some material and optical properties required for technological applications [3,29,30,31]. The most commonly observed crystalline phases of transition metal dichalcogenides are the 2H, 1T, 3R, and T_d_ phases, featuring, respectively, a hexagonal, trigonal, rhombohedral, and distorted octahedral symmetry of the crystal lattice [5,15,20,32].

Although several experimental and theoretical studies have been reported on AB_2_ systems in the solid state (i.e., on the bulk crystals, as well as on the layered WCh_2_ systems) [33,34,35,36], the basic physical chemistry of these systems in the gas phase is not yet well understood [37]. In the calculations of these systems in the solid-state, periodic boundary conditions are often invoked, and the properties reported are often limited to the geometry, lattice constants, band structure, density of states spectra, electron–hole recombination effect, exciton binding, and an analysis of stability; for example, [5,7,22,38,39]. Another focus was the determination of the nature of the dependence of the band dispersion on the mono-, bi-, and multi-layered WCh_2_ systems [35,36,40]. These are important properties of a solid-state material, and they assist one in predicting whether the material is suitable for synthesis, whether it is environmentally stable (in air, water, light, and atmospheric ambient temperature and pressure, etc.), and for applications in various areas of optoelectronics. 

The importance of transition metal dichalcogenide materials, and what appears to be an incomplete understanding of the nature of the chemical bonding in the interfacial region between the monolayers of the metal dichalcogenide bilayer and multi-layered systems, prompted this study. As in our recent report on MoCh_2_ systems [41], we focus here only on the dimers of the tungsten dichalcogenide series. We employ density functional theory (DFT) at the M06-2X level [42], with and without accounting for the effects of dispersion, in conjunction with three basis sets (LANL08, def2-TZVPPD, and Aug-cc-pVTZ(-PP) [43]).

The geometric, electronic, orbital, energetic, and charge-density based bond critical point and isosurface topologies of bonding interactions responsible for the formation of the three dimers, WCh_2_ (Ch = S, Se, Te), are discussed. (We use the term WCh_2_ rather than AB_2_ or ME_2_, in line with the IUPAC definition of chalcogen bonding (ChB) [44].) The orbital features are examined using natural bond orbital (NBO) analysis [45,46], whereas the charge-density-based features are examined using the quantum theory of atoms in molecules (QTAIM) [47], interaction region indicator (IRI) [48], independent gradient model (IGM) [49,50], and reduced density gradient (RDG) [51] approaches. We provide our views on whether or not the molecular electrostatic surface potential (MESP) method, often utilized to determine the active and inactive regions on molecular surfaces [52,53,54], is suitable for providing insight into the chemistry responsible for the attraction between Ch atoms of the isolated tungsten dichalcogenide monomer molecules leading to the formation of the dimers of tungsten dichalcogenides.

The intermolecular geometries that emerge from the gas phase calculations on (WCh_2_)_2_, and those known for the crystalline phase, are compared to address the titled concern: whether or not the chalcogen-centered non-covalent bonding interactions in (WCh_2_)_2_ dimers can serve as prototypes for a fundamental understanding of the local interfacial/interlayer chemical bonding environment in two-dimensional, layered tungsten dichalcogenides.

## 2. Results and Discussion

### 2.1. The Nature of the Interlayer/Interfacial Bonding Interactions in WCh_2_ Crystals

**Figure 2:**
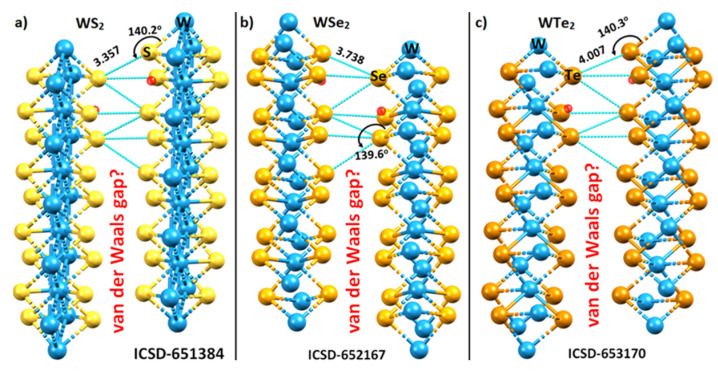
The nature of the interfacial (or interlayer) Ch···Ch bonding interactions between the metal bonded Ch (Ch = S, Se, Te) sites of the two monolayers in the 2H-WCh_2_ crystals: (**a**) WS_2_; (**b**) WSe_2_; and (**c**) WTe_2_. Selected Ch···Ch bond distances (Å) and ∠W–Ch···Ch bond angles (degrees) are shown. All Ch···Ch interlayer distances are equivalent for a given WCh_2_ crystal. The ICSD reference code is shown for each case. The so-called van der Waals gap between the chalcogenide layers is depicted in each occasion. Crystallographic axes are not shown.

The inter-layer contacts (dotted lines in cyan) between the tungsten bonded Ch sites in the WS_2_, WSe_2_, and WTe_2_ crystals of the 2H phase, in which the 4 × 1 × 1 supercells were used, are shown in Figure 2. The contacts are illustrated within the framework of the respective unit cells.

The S···S, Se···Se, and Te···Te inter-layer distances in the respective systems are either less than (for the former two) or slightly greater than twice the van der Waals radius of the respective chalcogen atom. For instance, the interlayer bond distances of 3.357 and 3.738 Å in WS_2_ and WSe_2_ are less than twice the vdW radius, 3.60 and 3.80 Å, of the S and Se atoms, respectively, whereas that of 4.007 Å in WTe_2_ is slightly longer than twice the vdW radius of Te, 4.00 Å (Bondi’s radius used: *r*_vdW_ (S) = 1.80 Å; *r*_vdW_ (S) = 1.90 Å; *r*_vdW_ (S) = 2.00 Å) [55]). From these distances alone, and since the bond distance is a measure of the bond strength, one might conclude that the Te···Te non-covalent interactions in WTe_2_ are weaker than the S···S and Se···Se bonds in WS_2_ and WSe_2_, respectively, and are of van der Waals type. Whether or not this immediate conclusion is misleading can only be verified once the energy of these interactions is calculated. We discuss this in a following section that summarizes the binding energy of the WCh_2_ dimers.

**Figure 3:**
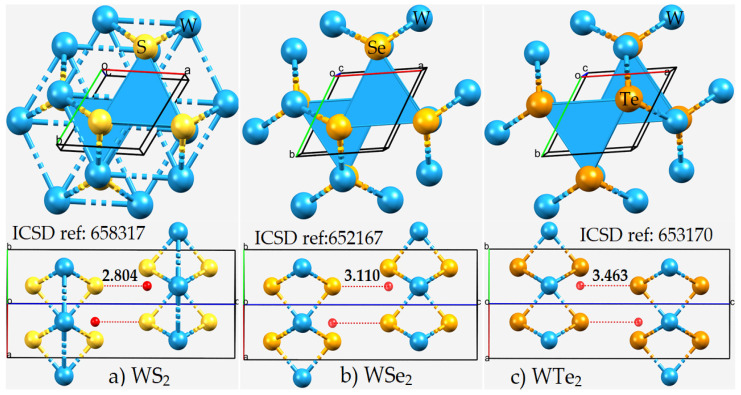
The top and side views of the unit-cells of (**a**) WS_2_, (**b**) WSe_2_, and (**c**) WTe_2_ in the 2H-phase, consisting of hexagonal triatomic layers, Ch–W–Ch, with a plane of metal atoms bound to two planes of chalcogen atoms (see Figure 1). The Inorganic Crystal Structure Database (ICSD) references to the structures are given. The distance between the W-bonded Ch site in an WCh_6_ unit in a monolayer and the centroid of the triangular face formed by three nearest Ch sites in the same unit in the neighboring layer (tiny red sphere) is shown in Å. Each structure is crystallized in the space group *P6*_3_*/mmc*.

The Ch···Ch non-covalent links between the monolayers in the WCh_2_ crystals are significantly bent, with ∠W–Ch···Ch = 140.2°, 139.6°, and 140.3° when Ch = S, Se, and Te, respectively. The non-linearity in these interactions could be a consequence of a relatively less negative Ch site in one monolayer interacting attractively with a relatively more negative junction region, delocalized over the triangular face formed by the three Ch sites, on the neighboring layer. This becomes evident if one examines the distance between the Ch site and the centroid of the triangular Ch···Ch···Ch face in the second monolayer. This distance is 2.804, 3.110, and 3.463 Å in WS_2_, WSe_2_, and WTe_2_, respectively (Figure 3), which is much smaller than the Ch···Ch inter-layer distances of the corresponding systems. Although these interactions are not linear and do not show up exactly along the W–Ch bond extensions, they could still be regarded as Ch···Ch bonded interactions. The validity of this view is put forward in the following section, where the nature of the electrostatic potential on the WCh_2_ surfaces is discussed.

The question that arises then is where is the van der Waals gap that is often referred to as existing between the monolayers in a bilayer 2H system of the WCh_2_ crystals? Does this purported van der Waals gap actually exist? The simple answer is that there is no such van der Waals gap between the monolayers in the 2H phase of the WCh_2_ crystal. The Ch atoms, bonded to W in the two W–Ch_2_ monolayers forming the bilayer 2H system, overlap with each other in the interface region. This means there is already a penetration between the Ch atoms in the interlayer region (vide infra). This overlapping is readily appreciated when one relates the Ch···Ch interlayer bond distance with the sum of the van der Waals radii of interacting Ch atomic domains. The Bondi’s van der Waals radii of S, Se, and Te are 1.80, 1.90, and 2.0 Å, respectively [55]. Therefore, twice of each of these radii gives values of 3.60, 3.80, and 4.0 Å, which are slightly smaller or comparable to the interlayer distances of 3.357, 3.738, and 4.007 Å observed in the WS_2_, WSe_2_, and WTe_2_ crystals, respectively. Since the van der Waals radii for all atomic domains of the periodic table are associated with an error of approximately 0.2 Å [56], it seems apparent that there is penetration between the Ch atoms in the interfacial/interlayer region of the WCh_2_ systems.

**Figure 4:**
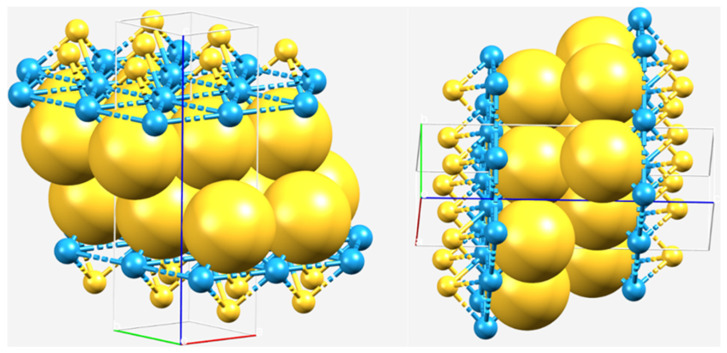
Illustration of combined ball-and-stick and space-filling views of the gapless WS_2_ crystal system in the 2H-phase. The tungsten-bonded S atoms (large yellow spheres) belonging to two different monolayers are “kissing” each other in the interface region, thus making the interfacial/interlayer region gapless. This may be likened to the concept of “kissing spheres” in coordination chemistry [57,58,59].

In the case of WTe_2_, the interlayer Ch···Ch bond distance in the crystal geometry is 4.007 Å and is within the radii error limit of twice the van der Waals radius of Te. This suggests that there is no van der Waals gap between the layers in either of the three crystal systems, as the Ch sites of the two interacting monolayers are bonded with each other through weak attractive forces (vide infra). That there is no physical gap (only voids) between the monolayers of WCh_2_ is also evident from the space-filling model shown in Figure 4, in which, the S atoms of each of the two monolayers are not only just facing each other, but “kissing” [57,58,59]. Further evidence of mutual penetration between the bonded atomic basins that emerges from a QTAIM analysis is discussed below.

### 2.2. The Nature of the Potential on the Electrostatic Surfaces of Isolated WCh_2_ Monomers

The MESP model [60] has been widely used to provide insight into the nucleophilic and electrophilic regions on the electrostatic surface of molecular entities [61,62,63,64,65,66]. Two key local descriptors of this model are *V_S,min_* and *V_S,max_*, the local most minimum and local most maximum of potential, respectively. Each can be positive or negative depending on the nature of the electron density depletion or accumulation at specific regions on the molecular surface [52,67,68]. When they are positive, the region is electrophilic, and when negative, it is nucleophilic [54,69,70]. These can show up on the same atom in a molecular entity; hence, the charge density on an atom in molecules may be anisotropic. Specifically, the local most maximum of potential, *V_S,max_*, on the surface of a bonded atom is commonly observed on the outer extension of a covalent bond, and the local most minimum of potential, *V_S,min_*, is commonly observed in a region dominated by π-density and lone-pair electron density [52,69] (i.e., around a covalently bonded atom in a molecule).

**Figure 5:**
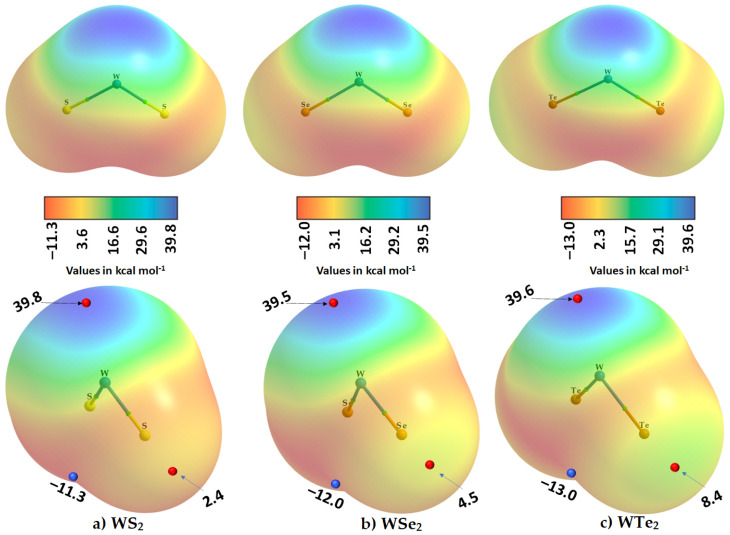
Two different views (top and bottom) of the 0.001 a.u. isodensity envelope mapped potential on the electrostatic surface of the isolated WCh_2_ monomers: (**a**) WS_2_, (**b**) WSe_2_, and (**c**) WTe_2_, obtained with M06-2X/Aug-cc-pVTZ(-PP). The tiny red and blue spheres represent the local most maximum and minimum of potentials (*V_S,max_* and *V_S,min_*), respectively. Superimposed in each case is the QTAIM molecular graph.

We used the 0.001 a.u. isodensity envelope, on which, we computed the potential on the electrostatic surfaces of the three WCh_2_ isolated monomers, the MESP graphs of which are shown in Figure 5. It is immediately evident that the surface of each isolated monomer comprises positive and negative regions; the charge density is indeed anisotropic in these species.

The most prominent negative region shows up on the surface of the junction region between the two Ch atoms in WCh_2_. The largest of these is found on the surface of the WTe_2_ molecule, with *V_S,min_* = −13.0 kcal mol^−1^ (Figure 5c).

The outer portions of W, opposite to the Ch–W bond axes, are very much positive (deep blue regions), which is not unexpected given that the metal center has empty, electron-deficient d_π_ orbitals. The principal reason for the emergence of the blue region on the surface of W is that the chalcogenides pull the electron density towards the bonding region from the tungsten atom, thus leaving behind a strongly positive region on the surface of the metal at equilibrium (a *σ*_d_ hole). The strength of the *σ*_d_ hole on W is very similar in the three monomers: 39.8 kcal mol^−1^ in WS_2_, 39.5 kcal mol^−1^ in WSe_2_ and 39.6 kcal mol^−1^ in WTe_2_, which explains why W accepts multiple chalcogen-centered bonds in the WCh_2_ crystals (see Figure 2 and Figure 3). This also explains why the region on the surface between the W and two Ch atoms is calculated to be the most negative (shown in red in Figure 5). 

By contrast, the axial portions of the Ch atoms bonded to tungsten are relatively more positive than the lateral portions that appear, firstly, along, and, secondly, around the W–Ch bond extensions. The first are nothing other than p-type σ-holes (*σ*_p_); they decrease in magnitude in the order WTe_2_ (8.4 kcal mol^−1^) > WSe_2_ (4.5 kcal mol^−1^) > WS_2_ (2.4 kcal mol^−1^), a trend that is consistent with the calculated binding energy of the WCh_2_ dimers (vide infra).

We did not observe a *V_S,min_* on the lateral sites of the bonded Ch atoms in WCh_2_. This does not mean that they are not present, or neutral; they would be observable if a higher isodensity envelope, for example 0.002 a.u., can be used for computing the electrostatic potential. For instance, a calculation with this isodensity envelope resulted in a *V_S,min_* of −2.1 kcal mol^−1^ for the lateral portion of S in WS_2_. Clearly, the Ch···Ch bonding features observed in the crystal, shown in Figure 2, are the result of attraction between sites of dissimilar charge densities localized on the lateral and axial sites of the bonded Ch atomic basins; this is nothing other than the attraction between a Lewis acid and a Lewis base site. While the Ch···Ch bonds are significantly bent, we still characterize them as chalcogen bonds that follow a Type I topology. (A Type I bonding topology is generally observed when the angle of interaction ∠D–Ch···A lies between 90° and 140°, where D is the donor fragment bonded with Ch, and A is the acceptor site [54]).

### 2.3. The Topologies of the Electron Localization and the Local Orbital Locator

We carried out an analysis of the electron localization function (ELF) [71] and the local orbital locator (LOL) [72,73], which are built using the kinetic energy density [74,75,76], since the nature of the electron localization and delocalization in WCh_2_ is one of the main aims of this study. This is necessary to gain insight into the nature of bonding and non-bonding pairs in these systems, as has been carried out elsewhere for other systems [77,78,79]. As Jacobsen summarized in his study [80] that followed on from the work of Schmider and Becke [72,73], a covalent bond is expressed in LOL as a local maximum between the bound centers in which the LOL = ½ surface enclosing the maximum has an increasing concave shape in multiple bonds. The LOL attains large values (above ½) in regions where the electron density is dominated by a single localized orbital. Lone-pairs are resolved as local maxima in the center of a characteristically shaped LOL = ½ surface. We invoked the LOL analysis to see whether it can adequately detect the localized pairs of electrons, bonding or non-bonding, that are not only evident in the topologies of ELF but also in the *L* (the negative of the Laplacian of the charge density, *L* = −∇^2^*ρ*) of QTAIM, since the latter is homeomorphic to the former, with few exceptions. While the LOL analysis encloses a large number of critical points, we restricted ourselves to the (3,−3) attractors in the gradient vector field of LOL. As Jacobsen noted previously [80], the critical point concept was borrowed from QTAIM, which refers to each nucleus as a (3,−3) attractor [47]. LOL also features such attractors in the bonding region, and in molecular regions that encloses lone-pairs [80].

Our calculations indicate that each W–S bond in WS_2_ is characterized by one (3,−3) attractor, which represents locally maximal electron localization, for which, the ELF and LOL values corresponding to this attractor are 0.766 and 0.626, respectively. In addition, each Ch atom of the monomer features a single attractor expected of the valence lone-pair, with ELF and LOL values of 0.889 and 0.621, respectively, in the case of WS_2_. The tungsten center in the monomers is surrounded with a quintet of lone-pair attractors; two of them are along the outer extension of the two Ch–W axes (LOL and ELF values for each are 0.890 and 0.737, respectively). For WSe_2_, the ELF (and LOL) values are 0.688 (0.581), 0.811 (0.494), and 0.888 (0.735) for the (3,−3) attractors responsible for the W–Se bonds, Se’s lone-pair, and W’s lone-pair along the Se–W bond extensions, respectively. For WTe_2_, these values are 0.645 (0.556), 0.748 (0.441), and 0.887 (0.734), respectively. Other than the valence shell lone-pair attractor around the Ch atom noted above, there are two other lone-pair attractors located in close vicinity to each Ch atom in WCh_2_ molecules. This suggests that the ELF and LOL models are superior to the MESP model in identifying the detailed topology of bonding and non-bonding pairs in this type of molecule.

While the W–Ch bonds in WCh_2_ have a multiple bond character (vide infra), the LOL picture still shows one attractor for each bond in the monomer. This mirrors the results in the study of Jacobsen, who found a single attractor for both C–C and C=C bonds, with a larger LOL for the former of 0.822 compared to 0.776; a value of 0.721 was found for the triple bond in N_2_ [80]. When acetylene was included in that study, the trend was maintained, and the LOL for C≡C was lower than that calculated for C–C and C=C. The increase in bond multiplicity led to an increase in the delocalized bonding character, and a reduction in the value of LOL. The trend in the ELF values for these bonds are very similar to that of LOL; ELF values of 0.9685 (for C–C single bond in C_2_H_6_), 0.9412 (for C=C double bond in C_2_H_4_), and 0.8906 (for C≡C triple bond in C_2_H_2_) have been reported [80,81]. This trend in the decrease in LOL and ELF with an increase in bond order is similar to what we observed for the monomers of the WCh_2_ series, where the LOL between the bonded atomic basins systematically decreased as one passes from WS_2_ through WSe_2_ to WTe_2_.

**Figure 6:**
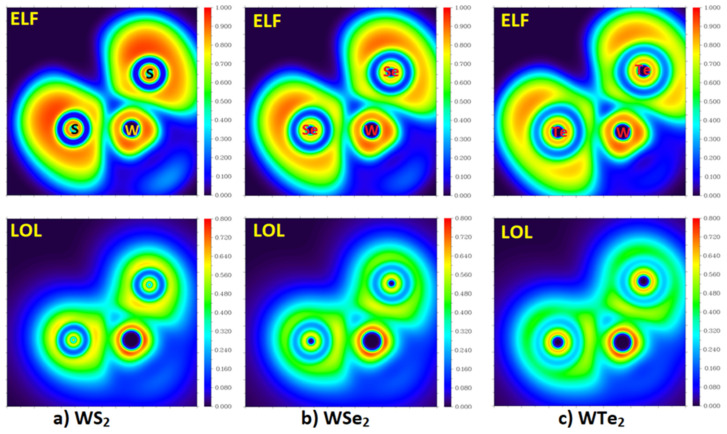
M06-2X/Aug-cc-pVTZ(-PP) level 2D maps of ELF and LOL functions (**Top** and **Bottom**, respectively) obtained on the plane defined by the two Ch atoms and one W atom in isolated WCh_2_ (Ch = S, Se, Te) monomers: (**a**) WS_2_; (**b**) WSe_2_; and (**c**) WTe_2_.

The 2D maps of the ELF and LOL functions for the molecular plane defined by the two Ch atoms and one W atom for the three WCh_2_ monomers are shown in Figure 6. Based on its definition, the ELF should range between 0 and 1 [71,79]. ELF = 1 corresponds to perfect localization and ELF = ½ corresponds to an electron gas. An ELF value close to 1 means that electrons are localized, implying a covalent bond, a lone pair, or inner shells of the atom involved. The value range of LOL is identical to ELF, but in the color bar in Figure 6 (bottom), the range is limited to between 0 and 0.8, corresponding to bonding and non-bonding attractor profiles. As can be seen from the 2D maps of ELF, the core regions of each atomic domain in each of the three monomers feature circular localization domains and have high ELF and LOL values close to 0.9. No strong localization is evident in the bonding regions between W and Ch in the three monomers, consistent with the multiple character of the W–Ch bonds.

### 2.4. Intermolecular Properties of (WCh_2_)_2_ Dimers, and Comparison with the WCh_2_ Crystals

**Figure 7:**
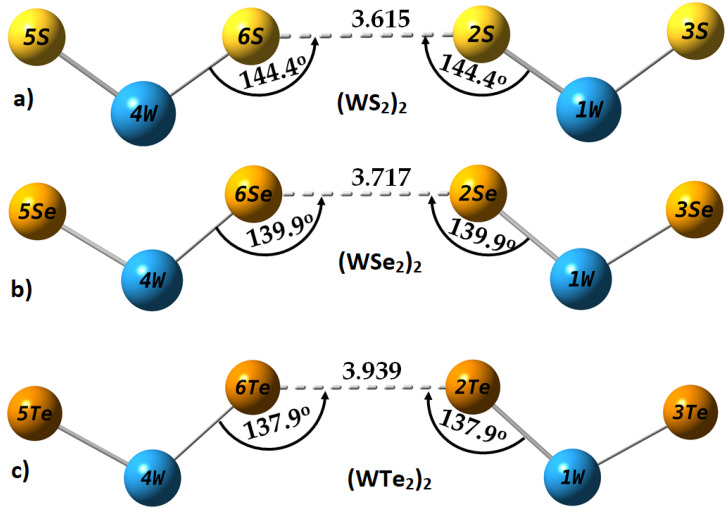
M06-2X/Aug-cc-pVTZ(-PP) computed fully relaxed geometries of (WCh_2_)_2_ (Ch = S, Se, Te) dimers, showing a Type I topology of non-linear Ch···Ch bonding links between the interacting monomers: (**a**) (WS_2_)_2_; (**b**) (WSe_2_)_2_; and (**c**) (WTe_2_)_2_. Selected bond lengths and bond angles are in Å and degrees, respectively. Atomic numbering and labeling are shown in each case.

The geometrically relaxed (WCh_2_)_2_ (Ch = S, Se, Te) dimers obtained with M06-2X/aug-cc-pVTZ(-PP) are shown in Figure 7. Table 1 summarizes the geometric, dipolar moment, and energetic properties of the three dimers, obtained using three different basis sets, including LANL08, def2-TZVPPD, and aug-cc-pVTZ(-PP). In addition, the corresponding results of M06-2X-D3/LANL08 are included, in which Grimme’s dispersion with the original D3 damping function was invoked to see the effect of dispersion on the properties of the dimers. As can be seen from Table 1, there are no remarkable changes in any of the properties when the effect of dispersion was incorporated with M06-2X. The comparison between M06-2X and M06-2X-D3 shows that, for the (WCh_2_)_2_ (Ch = S, Se) dimers, the Ch···Ch bond distances increased marginally by 0.003 Å, whereas, for (WTe_2_)_2_, the Te···Te bond distance decreased by 0.001 Å. The ∠W1–Ch2···Ch6 and ∠W4–Ch6···Ch2 angles increased by 0.2° for the first two dimers and remained unchanged for the latter. The ∆*E* and ∆*E*(BSSE) both increased by 0.1 kcal mol^−1^, yet no change was noticeable in the dipole moments of all three dimers. This indicates that the effect of dispersion on the dimer properties is very marginal when it is incorporated with the M06-2X functional.

Among the three basis sets tested, the best Ch···Ch intermolecular bond distances (when compared to the values obtained in the crystalline state, Figure 2) were obtained with the LANL08 functional. The def2-TZVPPD and Aug-cc-pVTZ(-PP) basis sets underestimate them to a small extent. However, the angles of the interaction, ∠W1–Ch2···Ch6 and ∠W4–Ch6···Ch2, are better reproduced by def2-TZVPPD and Aug-cc-pVTZ(-PP), and they are underestimated by LANL08. This is not surprising, since angles are more sensitive to the presence or absence of a d-polarization function in the basis set. We conclude that the Aug-cc-pVTZ(-PP) basis set performs best in obtaining reasonable estimates of *r*(Ch···Ch) and ∠W-Ch···Ch for all three (WCh_2_)_2_ dimers.

An inspection of the ∆*E* and ∆*E*(BSSE) values in Table 1 shows that the average BSSE energy is largest for the LANL08 basis set, and smallest with Aug-cc-pVTZ(-PP). This is why the ∆*E*(BSSE) is markedly smaller than ∆*E* for each of the three dimers with M06-2X/LANL08 and M06-2X-D3/LANL08. Nevertheless, the ∆*E*(BSSE) values obtained with the highest basis set applied suggest that the Ch···Ch interactions in the three (WCh_2_)_2_ dimers are of van der Waals type, since interaction energies of van der Waals complexes are generally close to, or below, −1.0 kcal mol^−1^, and are smaller than weak interactions in weakly bound complexes (interaction energies between −1.0 and −4.0 kcal mol^−1^) [82,83,84].

To determine the validity of this conclusion, we computed the binding energies of all three dimers using MP2(full), B97-D3(BJ), PW6B95-D3(BJ), and B3LYP-D3(BJ) using the M06-2X/Aug-cc-pVTZ(-PP) computed equilibrium geometries. The results are compared with Table 2. 

The values of ∆*E* and ∆*E*(BSSE) obtained with all of these four methods are larger than those computed with M06-2X and M06-2X-D3, suggesting that dispersion indeed plays an important role in elevating the complexation energies of the three dimers. The BSSE in energy is calculated to be very large with the post-Hartree–Fock method, MP2(full), and the trend in ∆*E* and ∆*E*(BSSE) across the dimer series is not systematic. That is not the case with all the DFT functionals used, which give a systematic increase in the bonding energy when passing from (WS_2_)_2_ to (WSe_2_)_2_ to (WTe_2_)_2_. This is also observed for ∆*E*(BSSE) as expected, given that Te is significantly more polarizable and less electronegative than Se, which, in turn, is more polarizable and less electronegative than S.

With the exception of M06-2X and M06-2X-D3, all other computational methods employed give ∆*E*(BSSE) close to, or larger than, −2.0 kcal mol^−1^ for (WTe_2_)_2_, showing that this dimer is not a van der Waals system. That may also be the case with (WSe_2_)_2_, since ∆*E*(BSSE) gives the predicted values of −1.16, −1.48, and −1.73 kcal mol^−1^ with PW6B95-D3(BJ), B97-D3(BJ), and B3LYP-D3(BJ), respectively. (MP2(full) predicts a spurious ∆*E*(BSSE) of −3.66 kcal mol^−1^, larger than that of (WTe_2_)_2_). By contrast, and despite the S···S intermolecular distance in (WS_2_)_2_ being significantly smaller than the Se···Se and Te···Te in (WSe_2_)_2_ and (WTe_2_)_2_, respectively (see Table 1 and Figure 2 for bond distances), the conclusion remains that (WS_2_)_2_ is a van der Waals system.

### 2.5. Charge Density Topological Properties of (WCh_2_)_2_ Dimers

**Figure 8:**
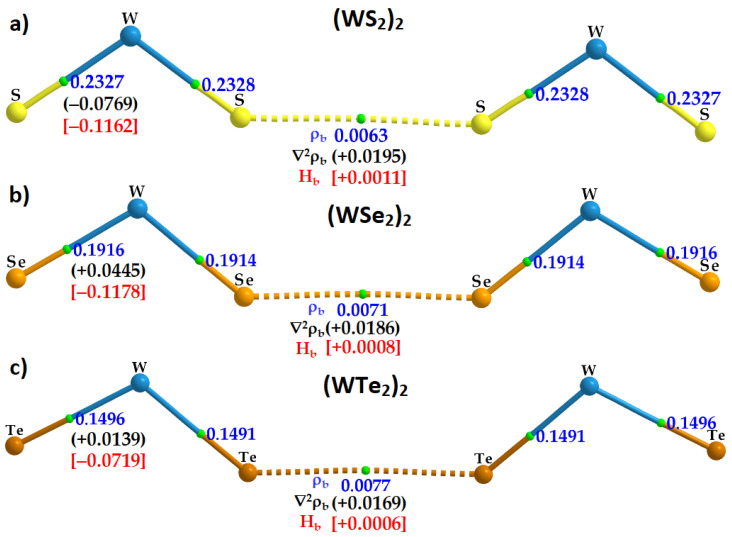
M06-2X/Aug-cc-pVTZ(-PP) computed QTAIM-based molecular graphs, presenting a description of intra- and inter-molecular interactions in WCH_2_ dimers: (**a**) (WS_2_)_2_; (**b**) (WSe_2_)_2_; and (**c**) (WTe_2_)_2_. Shown are the charge density (*ρ*_b_/a.u.; numbers in blue), the Laplacian of the charge density (∇^2^*ρ*_b_/a.u.; numbers in black), and the total energy density (*H_b_*/a.u.; numbers in red) at the Ch···Ch bond critical points. The former property is also depicted at the W–Ch bond critical points. Bond paths and bond critical points are shown as lines in atom color and tiny spheres in green between bonded atomic basins, respectively. Large spheres represent atomic domains for each dimer.

The QTAIM description of bonding interactions present in each (WCh_2_)_2_ dimer can be rationalized from the molecular graphs shown in Figure 8. As expected, they all show bond paths (line in atom color) and bond critical points (bcp, tiny spheres in green) between bonded atomic basins, indicative of chemical bonding between them [85]. QTAIM’s bond path descriptors do not just identify atom–atom links between molecular domains [86], but also allow for the recognition of various other interactions [87,88]; for example, between delocalized bonds in one molecular entity and the positive or negative site in another entity, as observed, for example, in Ti bonding to hydrocarbons [89].

The charge density *ρ*_b_ at the Ch···Ch bcp is smallest, 0.0063 a.u., for (WS_2_)_2_ and largest, 0.0077 a.u., for (WTe_2_)_2_. Interestingly, the trend in the *ρ*_b_ values is concordant with the BSSE-corrected binding energies calculated for the three (WCh_2_)_2_ dimers (see Table 1 and Table 2 for ∆*E*(BSSE)), suggesting that the charge density at the Ch···Ch bcp is indeed a measure of its bond strength.

**Figure 9:**
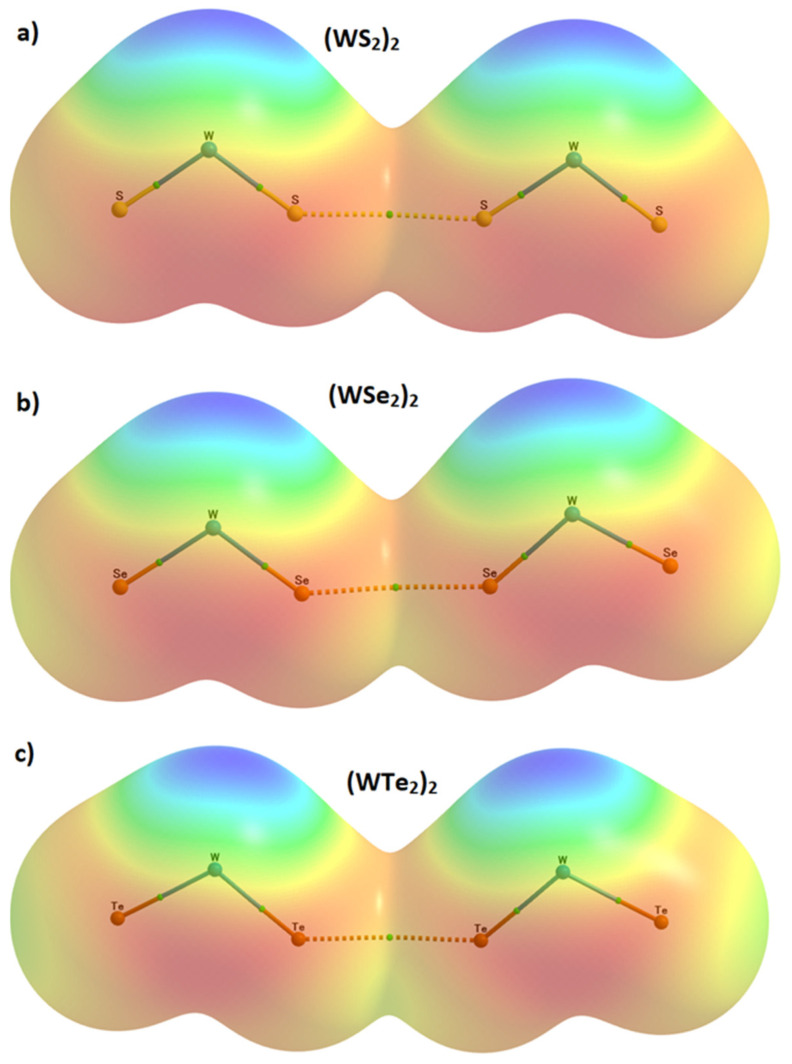
The 0.001 a.u. isodensity envelope mapped potential on the molecular surface of the three dimers, overlaid in the QTAIM bond paths and critical points, obtained using M06-2X/Aug-cc-pVTZ(-PP): (**a**) (WS_2_)_2_; (**b**) (WSe_2_)_2_; (**c**) (WTe_2_)_2_, suggesting no van der Waals gap existing between bonded chalcogen atoms in the intermolecular region since they are overlapped with each other. Color codes: red (−11.0 a.u.); yellow (+2.6 a.u.), green (+16.1 a.u.); cyan (+29.7 a.u.); and blue (+43.3 a.u.); 1 a.u. = 627.5 kcal mol^−1^.

Obviously, *ρ*_b_ at a Ch···Ch bcp is significantly smaller than that at a W–Ch bcp; the first are due to weak interactions between Ch atoms and the latter are the consequence of ionic bonds with some covalency. The small but non-zero values of *ρ*_b_ at the bcp of the Ch···Ch bonds are an indication of a bonded interaction signaled by QTAIM. The MESP model does not provide a way of quantifying the interaction. It does, however, enable one to see whether there is an atom–atom overlapping in the intermolecular region. This is indeed evident in Figure 9, which shows the way bonded atomic domains responsible for the formation of the (WCh_2_)_2_ dimers are linked with each other.

For quantum chemical systems in the gas phase, and within the framework of QTAIM, the van der Waals isosurface of a molecular entity is often defined as the *ρ* = 0.001 a.u. density envelope. The closest distance between a nucleus in a molecular entity and its van der Waals surface is called a “non-bonded atomic radius” [90]. Thus, for a non-covalently interacting pair of atoms A and B, the difference between the bond length of A···B and the sum of their non-bonded radii is regarded as the “mutual penetration distance” [91,92] between the van der Waals sphere of bonded atomic basins. It has been argued that this is a necessary and sufficient condition to identify hydrogen bonding in complex systems [93], and is transferable to other interactions. On this basis, in the case of (WS_2_)_2_, (WSe_2_)_2_, and (WTe_2_)_2_, the non-bonded radii of S2/S6, Se2/Se6, and Te2/Te6 atomic domains (see Figure 7) are calculated to be 2.134, 2.218, and 2.386 Å, respectively; twice these non-bonded radii are 4.268, 4.436, and 4.772 Å, respectively. Clearly, the difference between the calculated Ch2···Ch6 bond distance (see Table 1) and the twice non-bonded radius is 0.653, 0.719, and 0.834 Å for the (WS_2_)_2_, (WSe_2_)_2_, and (WTe_2_)_2_ dimers, respectively, suggesting that the mutual penetration between the two Ch sites responsible for the intermolecular region is strongest in (WTe_2_)_2_. Since the larger the mutual penetration distance between bonded atomic basins, the stronger the interaction between them, this accords with the trend observed in the binding energy of the dimers (Table 1 and Table 2). 

The sign of the Laplacian of the charge density, ∇^2^*ρ*_b_, at a bcp is often used to determine whether a bonding interaction is of a closed-shell (∇^2^*ρ*_b_ > 0) or open-shell (∇^2^*ρ*_b_ < 0) type [47,93]. A closed-shell interaction is observed in molecular entities that have non-covalent interactions associated with charge-density-depleted regions (for example, halogen bond, hydrogen bond, chalcogen bond, and pnictogen bond, van der Waals interaction); an open-shell interaction is synonymous covalent, polar covalent, or dative covalent bonds. A purely ionic bond—does such a thing exist? [94]—would be a closed-shell interaction. 

As expected, we found ∇^2^*ρ*_b_ > 0 at the Ch···Ch bcps of the (WCh_2_)_2_ dimers, with the largest value of 0.0195 a.u. for (WS_2_)_2_ and the smallest, +0.0169 a.u., (WTe_2_)_2_. ∇^2^*ρ*_b_ = −0.0769 a.u. at the W–Ch bcps of the (WS_2_)_2_ dimer, whereas ∇^2^*ρ*_b_ > 0 at the W–Ch bcps for the (WCh_2_)_2_ (Ch = Se, Te) dimers. This suggests that the W–S bonds in (WS_2_)_2_ are significantly more covalent than the W–Ch bonds in (WCh_2_)_2_ (Ch = Se, Te). That polar covalent bonds have ∇^2^*ρ*_b_ > 0 is not surprising [95,96], and our view on the polar covalent nature of the W–Ch bonds is in accordance with a similar conclusion arrived at for Mo–Ch bonds in MoCh_2_ crystals [97].

This conclusion above can be verified by considering the sign and magnitude of the total energy density, *H*_b_, which is the sum of the potential and gradient kinetic energy densities, *V*_b_ and *G*_b_, at the bcps. In QTAIM, when the potential energy density significantly dominates over the gradient kinetic energy density at the bond bcp, then the *H*_b_ < 0, which is indicative of an open-shell interaction. When the gradient kinetic energy density dominates at the bcp, *H*_b_ > 0, the signature of a closed-shell interaction is revealed.

Our results show that *H*_b_ > 0 for the Ch···Ch interactions in the three (WCh_2_)_2_ dimers; they are of the closed-shell type and can therefore be regarded as non-covalent interactions. On the other hand, the W–Ch bonds in the three dimers have *H*_b_ < 0, and become less negative in the order (WS_2_)_2_ > (WSe_2_)_2_ > (WTe_2_)_2_. These results are suggestive of W–Ch ionic bonds with a distinctive covalent character. Indeed, the combined signature ∇^2^*ρ*_b_ > 0 and *H*_b_ < 0 suggest a mixed ionic and covalent character that increases with a decrease in the electronegativity, and an increase in the polarizability of the chalcogen across the series from S through Se to Te.

The atomic charge is an informative quantum mechanical property of atoms in molecules [89] and crystals [98]. The results of our QTAIM calculations show that the integrated charge on each chalcogen site of the interacting WCh_2_ unit responsible for the dimer is negative; the charge on each of S, Se, and Te are −0.388, −0.240, and −0.075 *e* for (WS_2_)_2_, (WSe_2_)_2_, and (WTe_2_)_2_, respectively, with a decrease in negative charge paralleling the decrease in the electronegativity of the chalcogen. The corresponding charge on W is 0.782, 0.483, and 0.147 *e*, respectively. These are somewhat different from the values found for the same atoms in the isolated monomers (viz. 0.787 and −0.393 *e* on W and S in WS_2_; 0.485 and −0.243 *e* on W and Se in WSe_2_; and 0.146 and −0.073 *e* on W and Te in WTe_2_). Clearly, the dimer formation is accompanied by a rearrangement of charges, showing an effect of electrostatic polarization when the two monomers interact to form a dimer. Note that the magnitude of charges on the W and Ch atoms in the isolated monomers are substantially decreased compared to the formal charges +4.0 and −2.0 of W and Ch ions, respectively, showing that W becomes increasingly less positive, and Ch becomes increasing more positive. This implies that there is a significant transfer of charge from the W^4+^ ions to the Ch^2−^ ions during the formation of the isolated monomers. 

The rearrangement of charge under the circumstances given above has been noted in numerous occasions previously. For example, it was argued that M–Ch bonds in Mo-Ch_2_ crystals have significant covalent character, and a degree of polarity in the bond is caused by charge transfer from the metal to the chalcogen atoms [3,97,99,100]. In the case of WCh_2_, the electronegativity difference between W and Ch is 0.28, 0.10, and 0.07 for S, Se, and Te-containing systems, respectively; on this basis alone, one would conclude that the W–Ch bond is still a polar covalent bond, as has been deduced by others [101,102]. Although the two Ch atoms of the monomers that interact to form the dimer have the same charge, they still attract each other when forming the Ch···Ch bond. A similar conclusion can be arrived at by examining the nature of the electrostatic potentials of these interacting Ch atoms (vide supra). This reminds us of the concept of “like attracting like”—a concept that has been observed previously in a number of other systems [52,67,103,104,105]. 

A judgement on the polarity of a molecular domain can be made based on its dipole moment, *μ*. Our M06-2X/aug-cc-pVTZ(-PP) level calculation gave dipole moments of 2.73, 2.41, and 2.09 D for the monomers WS_2_, WSe_2_, and WTe_2_, respectively. Dimerization resulted in dipole moments of 5.30, 4.69, and 4.09 D for (WS_2_)_2_, (WSe_2_)_2_, and (WTe_2_)_2_, respectively. There is a clear enhancement of the dipole moment on the formation of the dimer, suggesting an increasing polarity of the dimers at equilibrium. 

### 2.6. Charge Density Based Isosurface Topologies of (WCh_2_)_2_ Dimers

A number of charge-density-based approaches have been developed within the last decade and applied to many chemical systems to explore chemical bonding interactions [48,49,50,51,106,107]. They have not only enabled the validation of graphically illustrated bonding environments obtained from isosurface topologies that depict the interaction between atomic domains in localized and delocalized bonds, and between delocalized bonds and anions, cations, or molecular entities, but have also greatly assisted a more rigorous characterization of chemical bonding interactions in general. Among these approaches are the RDG, IGM-δ*g*, DORI, and the recently proposed IRI-based NCI approach. Each has its limitations, and hence a joint application of these approaches to chemical systems may lead to a deeper understanding of the bonding involved between interacting atomic domains.

The RDG-based NCI characterization involves the plot of the sign(*λ*_2_) × *ρ* vs. RDG domains, where *λ*_2_ (*λ*_1_ ≤ *λ*_2_ ≤ *λ*_3_) is the second eigenvalue of the Hessian of the charge density matrix, and *ρ* is the charge density. RDG describes the reduced charge density gradient around the bond critical point region; the lower bound of RDG is zero and is reached whenever the charge density gradient vanishes at the bcp, a dimensionless quantity within the generalized gradient approximation of the exchange correlation term in DFT Hamiltonians [108,109]. 

The sign of *λ*_2_ can be positive or negative depending on whether it is associated with an eigenvector perpendicular to the bond path at a bcp, or whether it is associated with an eigenvector directed in the ring plane at a ring critical point [86]. Clearly, the sign of *λ*_2_ is one of the key parameters of the RDG-NCI approach, since it enables one to distinguish between supposedly attractive (*λ*_2_ < 0) and/or repulsive (*λ*_2_ > 0) interactions, and *ρ* quantifies the strength of that interaction. When the quantity sign(*λ*_2_) × *ρ* is mapped onto the RDG isosurface, both the nature and strength of the interactions within molecular entities become evident [51].

**Figure 10:**
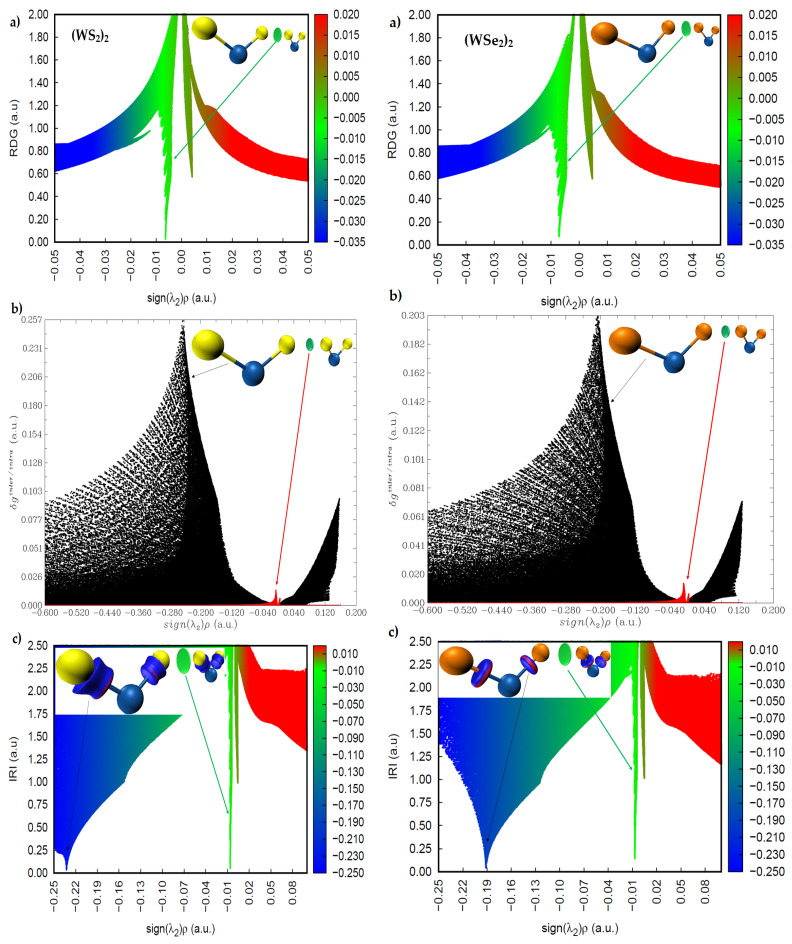
Comparison of the (**a**) sign(*λ*_2_) × *ρ* vs. RDG plot with those of the (**b**) sign(*λ*_2_) × *ρ* vs. IGM (δ*g^inter/intra^*) and (**c**) sign(*λ*_2_) × *ρ* vs. IRI plots for left, (WS_2_)_2_; right, (WSe_2_)_2_, obtained with M06-2X/Aug-cc-pVTZ(-PP). RDG, IGM(δ*g^inter^*^/*intra*^), and IRI isosurface domains were obtained with isovalues of 0.4, 0.01, and 1.0 a.u., respectively.

**Figure 11:**
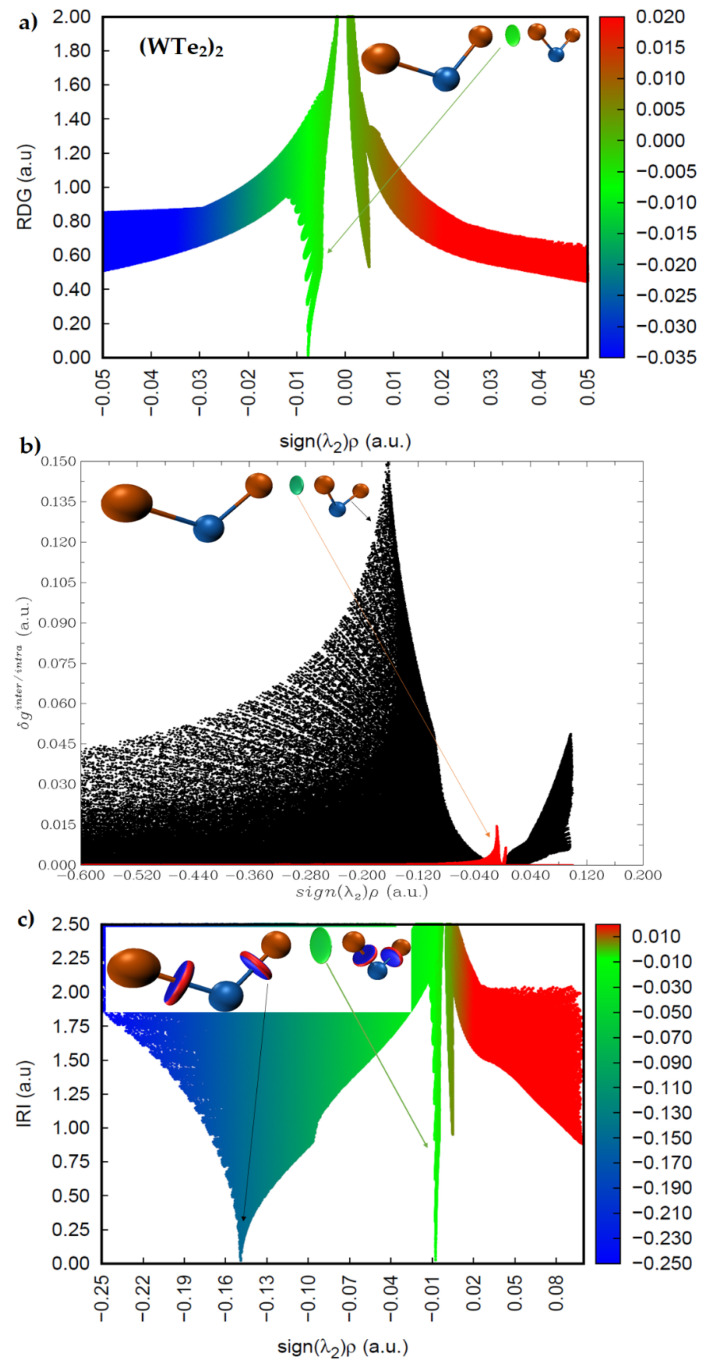
Comparison of the (**a**) sign(*λ*_2_) × *ρ* vs. RDG plot with those of the (**b**) sign(*λ*_2_) × *ρ* vs. IGM (δ*g^inter/intra^*) and (**c**) sign(*λ*_2_) × *ρ* vs. IRI plots for (WTe_2_)_2_, obtained with M06-2X/Aug-cc-pVTZ(-PP). RDG, IGM(δ*g^inter^*^/*intra*^), and IRI isosurface domains were obtained with isovalues of 0.4, 0.01, and 1.0 a.u., respectively.

The sign(*λ*_2_) × *ρ* vs. RDG plots are shown in Figure 10 and Figure 11 for (WS_2_)_2_, (WSe_2_)_2_, and (WTe_2_)_2_, respectively. The circular isosurface volume appearing between two Ch atoms (Ch6 and Ch2) in these dimers clearly corresponds to low-density regions, as shown by the greenish spikes in the sign(*λ*_2_) × *ρ* vs. RDG plots in the region −0.01 a.u. < sign(*λ*_2_) × *ρ* < 0.0 a.u. In this region, the sign(*λ*_2_) is negative. These are the characteristics of non-covalently bonded Ch···Ch interactions in all three dimers. There is a second spike in each of the three sign(*λ*_2_) × *ρ* vs. RDG plots, where sign(*λ*_2_) is positive. Since these appear in the low-density regions, with 0.0 a.u. < sign(*λ*_2_) × *ρ* < 0.01 a.u., these are signatures of van der Waals interactions.

One of the major drawbacks of RDG is that the isosurfaces of interacting fragments become noisy in large complex molecules; this makes it difficult to rationalize the actual molecular fragments or entities that are responsible for the development of specific RDG isosurface domains [49,50,107,110]. The IGM model was proposed to overcome this shortcoming. It has an advantage over the RDG-NCI approach in that it provides a way of identifying and quantifying the net ED gradient attenuation due to interactions using real or promolecular density [49,50].

A descriptor of the model, δ*g^inter^*^/*intra*^, can be derived; it uniquely defines inter- or intra-molecular interaction regions. Figure 10b and Figure 11b show the sign(*λ*_2_) × *ρ* vs. IGM(δ*g^inter^*^/*intra*^) for the three dimers. The spikes colored black are due to intramolecular interactions (W–Ch bonds), and those colored red are intermolecular interactions. The first appear in the high-density regions, and the second in the low-density regions. The IGM-based isosurface volumes that show up between the Ch atoms in (WS_2_)_2_, (WSe_2_)_2_, and (WTe_2_)_2_ correspond to the weak red spikes, and the strength of the interaction increases in this order: (WS_2_)_2_ < (WSe_2_)_2_ < (WTe_2_)_2_.

The more recently proposed IRI model [48] has an advantage over these two models above. It can not only provide insight into the presence of likely intermolecular interactions between the atomic domains, but can also give insight into the intramolecular interactions with the framework of isolated molecules. IRI relies on the charge density and its gradient to identify the inter- and inter-molecular interactions in molecular entities; IRI is simply the ratio between the charge density and its gradient norm. In other words, IRI is the gradient norm of the electron density weighted by the scaled charge density [48]. Figure 10c and Figure 11c show sign(*λ*_2_) × *ρ* vs. IRI for the dimers (WS_2_)_2_ and (WSe_2_)_2_, respectively, whereas that of the dimer (WTe_2_)_2_ is shown in Figure 11c. Two types of isosurfaces are evident. Those colored blue (dumbbell- and circular-disc-shaped) are a consequence of covalent bonding between W and Ch atomic basins. Those with a greenish color signal are the non-covalently bonded atomic basins of Ch atoms. Both isosurfaces were obtained with an IRI isovalue of 1.0 a.u., which is rather different to values of 0.4 and 0.01 a.u. used for RDG-NCI and IGM-δ*g* models, respectively, as each model requires a specific range of isovalues to generate isosurfaces of interest. The blue isosurfaces signify a significant accumulation of the charge density in the bonding region between W and Ch atomic basins, as expected for ionic bonding with some covalent character. The green isosurfaces indicate low-density regions, as similarly observed in the RDG and IGM-δ*g* plots that appear between the non-covalently bonded Ch atoms. The spikes in the sign(*λ*_2_) × *ρ* vs. IRI plots corresponding to these isosurfaces are identical to those of the sign(*λ*_2_) × *ρ* vs. IGM(δ*g^inter^*^/*intra*^) plots, indicating that both models provide a similar insight into the atomic basins causing the interactions and their strengths.

Klein and coworkers have recently proposed the intrinsic bond strength index (*IBSI*) that emerges from the IGM formulation [106]. According to them, each chemical interaction (or bond) in a molecular entity has its own IGM-δg*^pair^* signature, and, hence, the *IBSI* index. This means that the IGM and its *δg* descriptor are a way forward to locally quantify the electron density interpenetration from wavefunction calculations. These workers correlated the *IBSI* index with a number of conventional bond orders (such as Mulliken, Wiberg, Mayer, delocalization index, or electron localization function—ELF), albeit with poor regression coefficients, and called *IBSI* a new complementary index that is related to the bond strength. Considering A (as W1Ch3Ch2) and B (as W4Ch5Ch6) as two fragments of the (WCh_2_)_2_ dimer system (see Figure 7 atomic numbering), we calculated the *δg* indices for the three dimers that quantify the contribution of an atom–atom pair to the interaction between the two fragments.

Our calculations gave *δg* indices that were the largest at 0.159, 0.188, and 0.232 a.u., for each Ch2 and Ch6 atom in (WS_2_)_2_, (WSe_2_)_2_, and (WTe_2_)_2_, respectively, which are the pair of atoms that are closest to each other. Accordingly, the pair Ch2···Ch6 have the largest *δg* indices of 0.135, 0.163, and 0.209 a.u. for the corresponding systems, respectively, suggesting that the two Te atoms contribute largely to the Te···Te interaction in (WTe_2_)_2_ compared to that contributed by the two S and two Se atoms for the S···S and Se···Se interactions in (WS_2_)_2_ and (WSe_2_)_2_, respectively. Similarly, the sum of the related *IBSIW* (IBSI for weak interaction) for Ch2/Ch6 was 1.114, 1.258, and 1.413 a.u./Å^2^, respectively. The *IBSIW* indices for the Ch2···Ch6 pair were 1.034, 1.179, and 1.349 a.u./Å^2^, respectively, where *IBSIW* for any atom–atom pair *i* and *j* is defined by Equation (1) and Equation (2).

(1) $$IBSIW\left(i,\;j\right)=100\times \frac{\delta \;g_{i,\;j}}{d_{i,\;j}^{2}}$$

(2) $$\delta g_{i}=\underset{j}{\sum }\delta g_{i,j}\left(i\varepsilon A,\;j\varepsilon B\right)$$

where *d_i,j_* is the distance between atoms *i* and *j* in Å.

Since the larger the value of the *IBSIW* index, the stronger the interaction, it is clear that the preference of stability follows the order of (WS_2_)_2_ < (WSe_2_)_2_ < (WTe_2_)_2_, and is in accordance with the trend in the observed binding energies of these dimers (Table 2).

### 2.7. Second Order Charge Transfer between the Monomers Forming (WCh_2_)_2_ Dimers and Bond Order

Finally, we note that the attractive interaction between the WCh_2_ monomers leading to the formation of homodimers can indeed be recognized by the second-order estimates between donor and acceptor orbitals in an NBO basis. Specifically, our calculations suggest that there are several weak hyperconjugative charge transfer interactions between the interacting molecules forming the (WS_2_)_2_ dimers. The dominant delocalizations involve BD(3)W1–Ch2→BD*(3)W4–Ch6, BD(3)W4–Ch6→BD*(3)W1–Ch2, LP(2)Ch2/LP(2)Ch6→BD*(3)W4–Ch6/ BD*(3)W1–Ch2, where LP(2), BD(3), and BD*(3) represent the second π-type lone-pair, d_π_-type bonding, and anti-bonding-type orbitals, respectively. To give an example, the second order charge transfer delocalization energies *E*^(2)^ for these interactions were found to be 0.8, 0.8, and 0.5 kcal mol^−1^ for (WTe_2_)_2_, respectively. These results suggest that there are mutual hyperconjugative charge transfer interactions between the two monomers when they form a dimer. That W–Ch bonds in isolated WCh_2_ and complexed (WCh_2_)_2_ have the triple bond character, which was involved in the charge transfer delocalization, was evidenced by a bond order analysis [111,112,113,114]. For instance, the M06-2X/Aug-cc-pVTZ(-PP) computed Meyer; Fuzzy; and QTAIM bond orders are 2.8; 2.9; 2.6 and 0.05; 0.22; 0.115 for W–Te and Te···Te interactions in (WTe_2_)_2_, respectively. Very similar results were obtained for the W–Se and Se···Se interactions in (WSe_2_)_2_ and W–S and S···S interactions in (WTe_2_)_2_. The bond orders suggest that the link between W and Ch is of a triple-bond character, whereas that between the two Ch atoms (Ch···Ch) of the two interacting monomers forming the dimers is of a single-bond character. We note that the Mayer bond order [115] essentially reflects the number of electrons shared by two interacting atoms, which is very similar to QTAIM’s bond order. The latter has been called the delocalization index (the number of electrons exchanged between a pair of two atomic basins bonded to each other [50,111,116]).

## 3. Molecular Models

Three V-shaped WCh_2_ monomers and the three (WCh_2_)_2_ dimers were examined in this study using the DFT-M06-2X functional. The construction of the dimers was made possible by inspecting the geometry of the interfacial region between the WCh_2_ layers, reported in the crystals of WCh_2_ in the solid state [2,4,117,118].

Due to the fact that we were specifically interested in a basic understanding of the nature of the interactions between Ch sites in the interfacial/interlayer region of WCh_2_ crystals, we did not explore any other dimers in the conformational space. However, we are interested in exploring this conformational space to identify the global minimum for each dimer, and then to examine the extent to which the nature of the bonding environment and bonding energies between the two monomers changes upon dimerization; we will discuss this elsewhere. 

We have also limited our study to providing insight into the nature of the interfacial bonding in the 2H phase of the WCh_2_ crystals. This phase is semiconducting, and stable at room temperature. Reports show that another phase, the 1T phase, is highly unstable and converts back into the 2H phase with time and a variation in temperature. Metastable 1T(1T′) phases require higher formation energy compared to the thermodynamically stable 2H phase; thus, in standard chemical vapor deposition and vapor transport processes, the transition metal dichalcogenide materials normally grow in the 2H phases [15,119]. The crystal structure of the 2H phase of WS_2_ is shown in Figure 1.

## 4. Computational Methodology

Although transition metals in chemical systems often have multiconfigurational character [120], there have been several DFT studies [121,122,123] reported that utilized the M06-2X functional [42] for applications involving main-group thermochemistry, kinetics, noncovalent interactions, and electronic excitation energies to valence and Rydberg states. Wang et al. have demonstrated that the M06 and M06-2X hybrid metafunctionals have broad applicability, including transition metal systems [122]. As a result of this, we selected the M06-2X functional to model WCh_2_ monomers and (WCh_2_)_2_ dimers. We show that this functional reproduces the geometry of the intermolecular interactions found in the interfacial regions in the crystalline phase (cf. Figure 1 and Figure 2) very well. We selected three different basis sets (LANL08, def2-TZVPPD, and Aug-cc-pVTZ(-PP)—all available in the EMSL basis set exchange library [43]) to examine the extent to which the basis set size affects the intermolecular bond distances, bond angles, and other properties of the dimers examined. The basis set aug-cc-pVTZ(-PP) indicates that Aug-cc-pVTZ was centered on S and Aug-cc-pVTZ-PP was centered on Se, Te, and W. Tight convergence and ultrafine integration grids were chosen, recommended for DFT methods, and also the default option in Gaussian 16 [124]. The geometry optimization of each system was followed by a subsequent frequency calculation to identify the nature of the stationary or saddle points on the PES. The geometries of the monomers and dimers were all found to be true minima. 

The binding energy, ∆*E* (Equation (3)), for the (WCh_2_)_2_ dimers was calculated using the supermolecular procedure proposed by Pople [125]. ∆*E* was obtained by subtracting the sum of the total electronic energies (*E*^T^_sum_(monomers)) of the respective isolated monomers forming the dimers from the total electronic energy of each dimer (*E*^T^(dimer)). The energies of the monomers, as in the fully relaxed dimer configurations, were utilized. The basis set superposition error (BSSE) was calculated using the counterpoise procedure of Boys and Bernardi [126], Equation (4), where *E*(BSSE) is the BSSE energy.

∆*E* = (*E*^T^(dimer)) – (*E*^T^_sum_(monomers)) (3)

∆*E*(BSSE) = ∆*E* + *E*(BSSE) (4)

For reasons given in a following section, and because they are useful for the study of non-covalent interactions [127,128], we have also computed the BSSE-corrected ∆*E* of all three dimers using M06-2X-D3, MP2(full), B97-D3(BJ) [129], PW6B95-D3(BJ) [42,130], and B3LYP-D3(BJ) [131,132], where D3 is Grimme’s dispersion correction with the original D3 damping function [133], and D3(BJ) refers to the D3 version of Grimme’s dispersion with Becke–Johnson damping correction [130]. The fully relaxed M06-2X geometries of the dimers were used as the starting point for the latter five methods.

The topological properties (viz. the charge density, the Laplacian of the charge density and the total energy density at the bond critical points (*ρ*_b_, ∇^2^*ρ*_b_ and *H*_b_, respectively), the bond path, and the molecular graphs) were obtained using the AIMAll software [134]. The MESP graphs and the extrema of potentials were obtained using AIMAll and MultiWfn [110] software, respectively. The charge-density-based RDG [51], IRI [30], and IGM [50] isosurfaces that fingerprint intermolecular interactions between the monomers in the equilibrium geometries of the (WCh_2_)_2_ dimers were obtained using MultiWfn and VMD software [135], respectively. All of the electronic structure calculations were performed using Gaussian 16 [124].

## 5. Conclusions

In this study, we discussed the geometric, electronic, charge density topology, and energetic properties of (WCh_2_)_2_ (Ch = S, Se, Te) dimers examined at the M06-2X level of theory, in conjunction with three different basis sets: LANL08, def2-TZVPPD, and Aug-cc-pVTZ(-PP). We showed that, though each basis set has its own limitations, they all provided a very consistent Ch···Ch intermolecular bond distance for the three dimers examined and are well comparable with the interfacial Ch···Ch geometries observed for the WCh_2_ crystals in the room temperature 2H-phase. The overall performance in obtaining intermolecular bond distances and angles of approach leading to the formation of the three dimers is shown to be described well with the Dunning’s pseudopotential basis set Aug-cc-PVTZ(-PP). Although the M06-2X functional provided an intermolecular geometry in reasonable agreement with the experimentally established solid state geometry of WCh_2_, it underestimated the binding energies of the dimers. The effect of Grimme’s dispersion with the original D3 damping function had a negligible effect on the geometric, electronic, and energetic properties of the dimers, especially when it was incorporated with the M06-2X functional. However, the uncorrected and BSSE-corrected binding energies calculated on the M062X/Aug-cc-pVTZ(-PP) geometries of the dimers with MP2(full), B97-D3(BJ), PW6B95-D3(BJ), and B3LYP-D3(BJ) enabled us to demonstrate that (WSe_2_)_2_ and (WTe_2_)_2_ are weakly bonded dimers, and that (WS_2_)_2_ is a van der Waals system. That the (WS_2_)_2_ is a van der Waals system is a result of the attraction between the W-bound S atoms with positive electrostatic potentials along the W–S bond extensions in the isolated WS_2_ monomers, as has been observed for other systems before [52,84,103,104,105]. 

The QTAIM, IRI, IGM-δ*g^inter^*^/*intra*^, and RDG-based results associated with the charge density topological indicators have confirmed the occurrence of Ch···Ch attractive engagements leading to the formation of the (WCh_2_)_2_ dimers. The origin of the Ch···Ch interactions in (WCh_2_)_2_ was revealed by the second order hyperconjugative estimates of donor acceptor interactions in an NBO analysis, which showed that there are several weak charge transfer delocalizations between the interacting monomers at the dimer geometry, including, for example, LP(Ch)→BD*(3)W–Ch and BD(2/3)W–Ch→BD*(3)W–Ch. We therefore propose that the characteristics of the chalcogen–chalcogen bonding interactions observed in the (WCh_2_)_2_ dimers of tungsten sulfide, tungsten selenide, and tungsten telluride are prototypes for a basic understanding of the local interfacial/interlayer chemical bonding environment observed in the layered tungsten dichalcogenides in 2D, and that there is no van der Waals gap observed between the monolayers responsible for the interfacial/interlayer regions in bi-, tri-, and/or multi-layered tungsten dichalcogenides, but only voids.

## Figures and Tables

**Table 1:** M06-2X computed geometric, electronic, and energetic properties of (WCh_2_)_2_ (Ch = S, Se, Te) dimers, obtained with three different basis sets ^a,b^.

Method/Basis-Set	Dimer	*r*(Ch2···Ch6)	∠W1–Ch2···Ch6	∠W4–Ch6··Ch2	*μ*	∆*E*	∆*E*(BSSE)
M06-2X/LANL08	(WTe_2_)_2_	4.033	125.2	125.2	3.4	−1.07	−0.72
	(WSe_2_)_2_	3.766	136.4	136.4	5.3	−0.73	−0.43
	(WS_2_)_2_	3.603	147.6	147.6	6.6	−0.31	−0.09
M06-2X-D3 ^c^/LANL08	(WTe_2_)_2_	4.034	125.4	125.4	3.4	−1.18	−0.83
	(WSe_2_)_2_	3.769	136.6	136.6	5.3	−0.83	−0.53
	(WS_2_)_2_	3.602	147.6	147.6	6.6	−0.38	−0.17
M06-2X/def2-TZVPPD	(WTe_2_)_2_	3.939	138.0	138	4.2	−0.98	−0.85
	(WSe_2_)_2_	3.786	139.9	139.9	5.0	−0.47	−0.37
	(WS_2_)_2_	3.621	144.4	144.4	5.3	−0.23	−0.12
M06-2X/Aug-cc-pVTZ(-PP)	(WTe_2_)_2_	3.938	137.9	137.9	4.1	−0.98	−0.90
	(WSe_2_)_2_	3.717	139.9	139.9	4.7	−0.55	−0.43
	(WS_2_)_2_	3.615	144.4	144.4	5.3	−0.24	−0.12

^a^ See Figure 7 for atom numbering and labeling, and Figure 2 for comparison with the interlayer geometry of the WCh_2_ (Ch = S, Se, Te) crystals. ^b^ Geometric properties included the Ch···Ch intermolecular bond distance (Å) and the ∠W-Ch···Ch bond angles (in degree); the electronic property included the dipole moment μ (in Debye); and the energetical properties included the uncorrected and BSSE corrected binding energies ∆*E* and ∆*E*(BSSE), respectively (in kcal mol^−1^). ^c^ Grimme’s dispersion with the original D3 damping function.

**Table 2:** Computed uncorrected and BSSE-corrected binding energies (∆*E* and ∆*E*(BSSE), respectively) of the (WCh_2_)_2_ (Ch = S, Se, Te) dimers, obtained with several methods in conjunction with the Aug-cc-pVTZ(-PP) basis set ^a^.

Dimer	M06-2X		M062X-D3 ^b^		MP2(Full)		B97-D3(BJ) ^c^		PW6B95-D3(BJ) ^c^		B3LYP-D3(BJ) ^c^	
	∆*E*	∆*E*(BSSE)	∆*E*	∆*E*(BSSE)	∆*E*	∆*E*(BSSE)	∆*E*	∆*E*(BSSE)	∆*E*	∆*E*(BSSE)	∆*E*	∆*E*(BSSE)
(WTe_2_)_2_	−0.98	−0.90	−1.09	−1.01	−5.63	−3.32	−2.17	−2.00	−2.05	−1.94	−2.65	−2.52
(WSe_2_)_2_	−0.55	−0.43	−0.65	−0.54	−5.85	−3.66	−1.48	−1.37	−1.26	−1.16	−1.82	−1.73
(WS_2_)_2_	−0.24	−0.12	−0.32	−0.20	−4.16	−2.95	−0.92	−0.80	−0.81	−0.70	−1.25	−1.14

^a^ ∆*E* and ∆*E*(BSSE) in kcal mol^−1^. ^b^ D3 version of Grimme’s dispersion with the original D3 damping function incorporated. ^c^ D3 version of Grimme’s dispersion with Becke–Johnson damping incorporated.

## Data Availability

This research did not report any data.
